# Relationship of Tumor-Associated Macrophage Population Detected by CD68 PG-M1, CD68 KP1, and CD163 with Latent EBV Infection and Prognosis in Classical Hodgkin Lymphoma

**DOI:** 10.5146/tjpath.2020.01514

**Published:** 2021-05-15

**Authors:** Hanife Seda Mavili, Aydın Isisag, Ayca Tan, Mine Miskioglu, Lale Saka Baraz, Nalan Nese

**Affiliations:** Department of Pathology, Manisa Celal Bayar University, Faculty of Medicine, Manisa, Turkey; Department of Hematology, Manisa Celal Bayar University, Faculty of Medicine, Manisa, Turkey; Department of Internal Medicine, Manisa Celal Bayar University, Faculty of Medicine, Manisa, Turkey

**Keywords:** Classical Hodgkin Lymphoma, Macrophages, Epstein-Barr virus, CD68, CD163

## Abstract

***Objective:*** To evaluate the quantity of tumor-associated macrophages (TAMs) in cases of Hodgkin Lymphoma of classical type (cHL), and to reveal possible associations between TAM intensity and latent Epstein-Barr virus (EBV) infection, overall survival, progression-free survival, prognostic indices, and clinicopathological parameters.

***Materials and Methods: *** A total 46 cases of cHL with complete clinical records were selected and re-evaluated histopathologically. Staining for CD68 (PG-M1; KP1 clones) and CD163 was evaluated and the cut-off values were defined. Also, all cases were evaluated using the chromogen in situ hybridization (CISH) method with EBER (Epstein-Barr virus-encoded RNA) probes for the presence of possible EBV infection.

***Results: *** It was found that high expression levels of PG-M1 and high International Prognostic Scores (IPS) were associated with shortened overall survival (p=0.047, p=0.013). Cases with 2 or less areas of nodal region involvement were observed to have longer progression-free survival period (p=0.043). Higher expression levels of CD68 PG-M1, CD68 KP1, and CD163 were found to show significant associations with the presence of some clinical parameters such as the presence of B symptoms, spleen involvement, and the presence of EBV infection.

***Conclusions: *** Our findings suggest that increase of PG-M1+ TAM is associated with shortened overall survival, while higher expressions of all immunohistochemical markers are statistically significantly associated with the presence of EBV infection and clinical parameters mentioned above. These findings indicate that highlighting the TAM rate via macrophage markers in cases of cHL could be helpful in determining the prognostic risk groups and the relevant results should be mentioned in pathology reports.

## INTRODUCTION

Hodgkin Lymphoma (HL) makes up 0.6% of all malignancies and 10% of lymphomas (1), and differs from other malignant neoplasms for not only by its clinical presentation but also because it contains very few neoplastic cells (Hodgkin/Reed Sternberg (HRS) cells) in an inflammatory cell-rich background (2). In recent years, it has been considered that this inflammatory background is an important factor in tumor progression and prognosis in some hematologic malignancies such as follicular lymphoma and Hodgkin Lymphoma of the classical type (cHL) (3–5). To highlight prognostic factors and risk groups, many studies have been performed up to now by different teams, such as the German Hodgkin Lymphoma Study Group (GHSG), the European Organization for Research and Treatment of Cancer (EORTC), the National Cancer Institute of Canada (NCIC), and the National Comprehensive Cancer Network (NCCN). Although the international prognostic score (IPS) still remains important to predict the course of the disease (6,7), novel markers are needed to govern modern therapeutical methods in a more exact way. It has been revealed that reactive inflammatory cells in the microenvironment of the involved tissue enhance the proliferative capacity and anti-apoptotic features of HRS cells, and there has been an increase in the number of studies on biological markers since the1980s. First, an abundance of TAM in tumoral tissue was shown to be associated with B symptoms and a worse prognosis (8). While some of the other studies dealing with the same issue have claimed that an increase in the number of TAM causes a decrease in overall survival and could be used as a new marker in evaluating risk groups (4,5), some others found no relationship between these two parameters (9,10). Also, there are studies showing a correlation between TAM intensity and EBV positivity of the neoplastic cells (4,11). There seems to be an obvious need for other studies for these findings to take their place in routine practice. In this study, two clones of the CD68 antibody, the most commonly used monocyte /macrophage marker, and CD163 antibody, a more specific marker than CD68, were used to assess the intensity of TAM in the tumoral microenvironment. An attempt was then made to statistically determine the possible associations between TAM intensity and prognosis and latent EBV infection. It was also aimed to determine whether there are significant associations between TAM intensity and IPS, EORTC score, and clinicopathologic parameters.

## MATERIAL and METHODS

Forty-six cHL patients, who had been regularly followed-up between 2008 and 2016 at the Hematology Department of Manisa Celal Bayar University Hospital, and whose slides and paraffin blocks were extracted from the archives of the Department of Pathology, were evaluated in the study. The study was approved by the institutional ethics committee (Date: 25.02.2016, Reference No: 20478486-51)

Among these, 24 were nodular sclerosing (NS), 20 were mixed cellularity (MS), 1 was lymphocyte rich (LR), and 1 was the lymphocyte depleted (LD) type. Course of disease, clinical features, laboratory findings, presence or absence of B symptoms, presence or absence of bulky (R ≥ 10 cm) lesion(s), presence or absence of mediastinal mass, stage of the disease according to the modified Ann Arbour System, and presence or absence of recurrence(s) and progression were noted from the patients’ files and pathology reports. Risk groups were determined according to IP score and EORTC score. As the number of cases is not high, both scoring systems were applied for all patients at any stage. Patients were divided into two categories according to IP scores; low-risk group (IPS≤ 3) and high-risk group (IPS≥4). Another categorization was also performed according to the EORTC score; low-risk group (not bearing any of the above-mentioned risk factors) and high-risk group (bearing at least one risk factor).

### Treatment

From the patients’ files, it was determined that various treatment protocols had been administered depending on the stage of the disease and the performance of the patients. Forty-four of the cases underwent a conventional adriamycin, bleomycin, vinblastine, dacarbazine (ABVD) treatment regimen, and in addition to this, radiotherapy was applied to the tumor-associated area in some of the cases. Because the performance status of the remaining two cases was low, no chemotherapy was administered and they died 2 months and 8 months after the initial diagnosis. One of these patients had the LD and the other had the MS type of cHL, respectively. In 9 out of 18 cases showing recurrence or progression after the initial therapy, additional chemotherapy regimens were also administered.

### Immunohistochemistry and chromogenic in situ hybridization

For immunohistochemistry, 4μ-thick sections obtained from formalin-fixed/paraffin-embedded tissues were placed on positive-charged electrostatic slides (Isotherm Technical Laboratory Glass Materials). All sections were then placed on a fully automated immunohistochemical staining machine (Ventana, Benchmark, XT IHC/ISH). UltraView Universal DAB Detection Kit compatible with the device was used for IHC staining. Monoclonal mouse primary antibodies CD163 (clone MRQ-26, prediluted, VENTANA, Cat. No: 760-4437) and CD68 (clone KP-1, prediluted, VENTANA, Cat. No: 790-2931and clone PG-M1, prediluted, DAKO, Code: IS613, prediluted, Cat. No: 760-4437) were applied and staining was completed according to the standard procedures. To detect EBV-RNA with an EBER (Epstein-Barr virus-encoded RNA) probe via the CISH method, 4μ-thick tissue sections placed on positive-charged electrostatic slides were also used. All tests were performed with a fully automated ISH machine (Ventana, Benchmark XT, IHC/ISH), by using EBER 1 DNP Probe (Regulatory status: ASR; Cat. No: 760-1209), compatible with the machine, and the Ultraview AP Red ISH Kit for EBV RNA signaling, and staining was completed according to the standard procedures.

### Evaluation of immunohistochemical and ISH staining

For immunohistochemical studies, the rate of staining macrophages was determined via consensus of two observers (A. I. and H. S. M.) by standard research microscope. To assess the rate of staining macrophages, microscopic fields that were rich in HRS cells were chosen and almost the same fields were used to evaluate all three antibodies. Assessment was made in 5 neighboring high-power fields in each case in a quantitative manner, by counting the number of staining macrophages and all other non-neoplastic cells, and recording the rate of staining macrophages in multiples of 5%. To assess a case as EBER (+), it has been stated that almost all neoplastic cells have to be stained with EBER-ISH technique (12). So, cases with nuclear staining of nearly all neoplastic cells were assessed to be positive with EBER.

### Statistical Analysis

As the highest expression levels were obtained with CD163 followed by CD68 PG-M1 and CD68 KP1 expressions in a decreasing manner, the rates of 50%, 40% and 30%, which were close to the median values were considered as primary cut-off values, respectively. Furthermore, the rate of 30% for CD163 and 10% for CD68 PG-M1 and CD68 KP1 were determined as secondary cut-off values in order to investigate whether lower expressions were significant. In survival analysis, overall survival was defined as the period from diagnosis to death of the patient for any reason or to last recorded follow-up date while progression-free survival was defined as the period from diagnosis to progression or recurrence of the disease or to last recorded follow-up date. To assess possible relations between the above-defined cut-off values and the clinicopathologic parameters, laboratory findings, and the presence of EBV, Fisher’s exact test was used. Significant variables obtained in univariate analysis were then evaluated for multivariate analysis by using the logistic regression model. The Kaplan-Meier procedure was used for survival analysis and the log-rank test was applied to compare survival curves. Pearson and Spearman correlation tests were utilized to evaluate the correlation between expressions of the three immunohistochemical markers. P values smaller than 0.05 were considered as statistically significant while p values equal or greater than 0.05 but smaller than 0.1 were considered as having borderline significance. All tests were performed by using SPSS (Statistical Package for Social Sciences), version 20.0.

## RESULTS

### Age, gender, and clinicopathologic findings

Age, gender and clinicopathologic findings of all cases are shown in Table 1. A total of 5 patients died at 2, 6, 8, 40 and 50 months after the initial diagnosis, respectively.

**Table 1:** Age, gender, clinicopathologic findings of the cases and relationship of survival with clinicopathologic parameters and expression levels of histiocytic markers according to two different cut-off points.

**Parameters**	**Mean overall survival period (months**	P value for overall survival period*	**Mean progression-free survival period (months)**	P value for progression-free survival period*	** Parameters**	**Mean overall survival period (months)**	P value for overall survival period*	**Mean progression-free survival period (months)**	P value for progression-free survival period*
**Age** <50 (n=30) **≥50 (n=16)**	77 72	0.518	49 53	0.707	**Stage** Early(I-II) (n=18) Advanced (III-IV) (n=28)	* * *83* *63*	**0.090**	62 37	0.104
**Gender** Female (n=23) Male (n=23)	78 68	0.174	42 56	0.308	**Subtype** NS (n=24) MS (n=20) (LR (n=1) and LD (n=1) were not evaluated)	* * *79* *69*	0.235	49 49	0.776
**B symptoms** Present (n=23) Absent (n=23)	71 77	0.432	46 53	0.756	**EORTC** Low risk (n=11) High risk (n=35)	** 64	**0.076**	58 44	0.463
**Bulky lesion** Present (n=8) Absent (n=38)	** 75	0.375	25 51	0.998	**IPS** 0-3 (n=39) ≥4 (n=7)	79 17	**0.013**	51 16	0.746
**Extralymphatic involvement** Present (n=20) Absent (n=26)	38 81	0.067	25 59	**0.051**	**Progression/recurrence** Present (n=18) Absent (n=28)	41 81	** ** **0.047**		
**Mediastinal mass** Present (n=5) Absent (n=41)	** 76	0.523	28 49	0.525	**Hodgkin/Reed-Stenberg cells** Rare (n=8) Medium/abundant (n=38)	79 54	0.310	41 50	0.620
**Bone marrow involvement** Present (n=12) Absent (n=34)	** 74	0.277	30 53	0.554	**EBER** Positive (n=27) Negative (n=19)	73 74	0.304	61 36	**0.062**
**Splenic involvement** Present (n=16) Absent (n=30)	71 77	0.426	53 49	0.898	**CD68 PG-M1** >40%; ≤40% >10%; ≤10%	58; 85 74; **	** ** **0.047** 0.293	46; 52 55; 29	0.834 0.205
**Number of involved nodal regions** 1-3 (n=20) 4-6 (n=12) ≥7 (n=14)	80 35 63	0.541	64 15 37	**0.043**	**CD68 KP1** >30%; ≤30% >10%; ≤10%	43; 79 71; **	0.300 0.127	32; 51 57; 32	0.947 0.124
**Sedimentation rate (mm/h)** >50 (n=17) ≤50 (n=29)	45 81	**0.073**	29 58	**0.090**	**CD163** >50%; ≤50% >30%; ≤30%	75; 75 70; 76	0.990 0.127	49; 50 57; 41	0.881 0.412

**NS:** Nodular sclerosing, **MS: **Mixed cellularity, **LR:** Lymphocyte rich, **LD:** Lymphocyte depleted, **IPS:** International Prognostic Score, **EORTC:** European Organization for Research and Treatment of Cancer, **EBER:** Epstein-Barr virus-encoded RNA. *Log-rank test, **mean overall survival period (months) was not computed since no deaths (events) occurred in this category.

### Evaluation of light microscopy, CD163 and CD68 expressions and CISH studies

**Figure 1:**
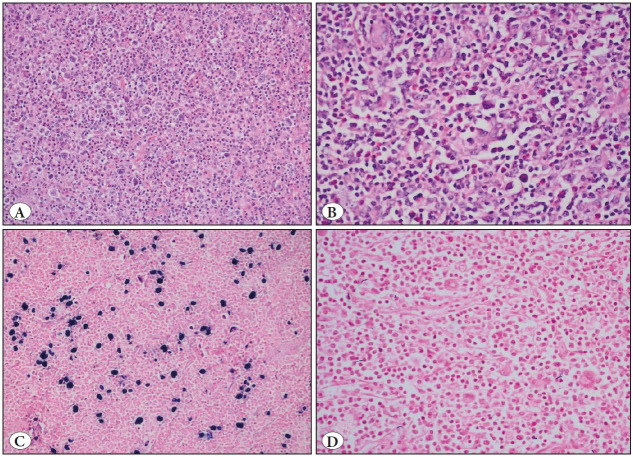
**A)** Abundant HRS cells in case #42 (H&E; x200). **B)** Scant HRS cells in case #18 (H&E; x400). **C)** EBER ISH positive HRS cells in case #42 (CISH; x200). **D)** EBER ISH negative HRS cells in case #18 (CISH; x200).

**Figure 2:**
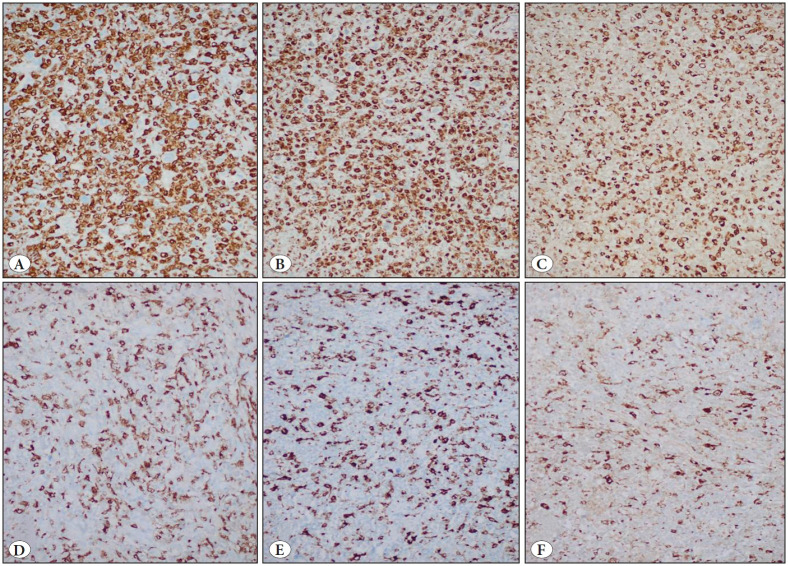
**A)** 70% CD163 expression in case #42(IHC; x200). **B)** 65% CD68 PG-M1 expression in case #42 (IHC; x200). **C)** 45% CD68 KP1 expression in case #42 (IHC; x200). **D)** 10% CD163 expression in case #18 (IHC; x200). **E)** 10% CD68 PG-M1 expression in case #18 (IHC; x200). **F)** 5% CD68 KP1 expression in case #18 (IHC; x200).

Examples of abundant and scant HRS cell population in the non-neoplastic background in H&E stained slides of two different cases are shown in Figure 1B, respectively. In these cases, EBER ISH was positive in the first (Figure 1) and negative in the second (Figure 1). When TAM intensity was evaluated in all three markers, it was seen that the highest levels of expression were observed with CD163 and lower levels were detected with CD68 clone KP1. Examples of high and low expression levels of CD163, CD68 PG-M1, and CD68 KP1 of the above cases are presented in Figure 2 and Figure 2, respectively. However, expression levels of all three markers were correlated statistically (p<0.001; Pearson and Spearman correlation tests). It was also observed that staining with histiocytic markers was more intense in the areas where neoplastic cells were abundant, especially with CD163.

### Relationship of CD163 and CD68 expression, EBV infection, age, gender and clinicopathologic parameters with survival

Statistical data obtained to assess relationship of survival with CD163 and CD68 PG-M1 and CD68 KP1 expressions according to two different cut-off points, EBV infection, age, gender and clinicopathologic parameters are shown in Table 1. It was stated that overall survival was decreased with higher CD68 PG-M1 expression levels (according to the 40% cut-off point) and higher IP scores (p=0.047 and p=0.013, respectively). There was a significant relationship between a long duration of progression-free survival and the number of involved nodal regions below 3 (p=0.043), while there was a borderline statistically significant difference with the presence of EBV infection (p=0.062). No significant differences were found between survival and types of cHL (i. e. NS or MS types; LR and LD types were not analyzed).

### Relationship of CD163 and CD68 expression with EBV infection and clinicopathologic parameters

Statistical data obtained to assess relationship of CD163, CD68 PG-M1, and CD68 KP1 expressions with EBV infection and clinicopathologic parameters are shown in Table 2. Increased staining intensity for all three histiocytic markers was found to be correlated with the presence of B symptoms, splenic involvement, presence of EBV infection, and high-risk group according to EORTC. Multivariate analyses revealed that significant relationships between all three markers and EBV infection and B symptoms were retained. This was also the same for increased CD163 and CD68 PG-M1 expressions and splenic involvement.

**Table 2:** Significant **(bold)** and borderline significant (red) results obtained between clinicopathologic parameters and expression levels of CD68 PG-M1 according to 40% and 10% cut-off points, CD68 KP1 according to 30% and 10% cut-off points, and CD163according to 50% and 30% cut-off points.

	**CD 68 PG-M1**						**CD68 KP1**						**CD 163**					
	**40% cut-off point**			**10% cut-off point**			**30% cut-off point**			**10% cut-off point**			**50% cut-off point**			**30% cut-off point**		
**Parameters**	**>40%** **(n=18)**	**≤40%** **(n=28)**	**p**	**>10%** **(n=38)**	**≤10%** **(n=8)**	**p**	**>30%** **(n=12)**	**≤30%** **(n=34)**	**p**	**>10%** **(n=30)**	**≤10%** **(n=16)**	**p**	**>50%** **(n=12)**	**≤50%** **(n=34)**	**p**	**>30%** **(n=22)**	**≤30%** **(n=24)**	**p**
**Age** **<50 (n=30)** **≥50 (n=16)**	10 (33.3%) 8 (50%)	20 (66.7%) 8 (50%)	0.215	23 (76.7%) 15 (93.8%)	7 (23.3) 1 (6.2%)	0.147	6 (20%) 6 (37.5%)	24 (80%) 10 (62.5%)	0.174	17 (56.7%) 13 (81.3%)	13 (43.3%) 3 (18.7%)	**0.088**	6 (20%) 6 (37.5%)	24 (80%) 10 (62.5%)	0.174	10 (33.3%) 12 (75%)	20 (66.7%) 4 (25%)	**0.008**
**Gender** **Female (n=23)** **Male (n=23)**	9 (39.1%) 9 (39.1%)	14 (60.9%) 14 (60.9%)	0.618	16 (69.6%) 22 (95.7%)	7 (30.4%) 1 (4.3%)	**0.023**	7 (30.4%) 5 (21.7%)	16 (69.6%) 18 (78.3%)	0.369	12 (52.2%) 18 (78.3%)	11 (47.8%) 5 (21.7%)	**0.060**	5 (21.7%) 7 (30.4%)	18 (78.3%) 16 (69.6%)	0.369	10 (43.5%) 12 (52.5%)	13 (56.5%) 11 (47.8%)	0.384
**B symptoms** **Present (n=23)** **Absent (n=23)**	13 (56.5%) 5 (21.7%)	10 (43.5%) 18 (78.3%)	**0.017**	22 (95.7%) 16 (69.6%)	1 (4.3%) 7 (30.4%)	**0.023**	10 (43.5%) 2 (8.7%)	13 (56.5%) 21 (91.3%)	**0.008**	21 (91.3%) 9 (39.1%)	2 (8.7%) 14 (60.9%)	**<0.001**	11 (47.8%) 1 (4.3%)	12 (52.2) 22 (95.7)	**0.001**	15 (65.2%) 7 (30.4%)	8 (34.8%) 16 (69.6%)	**0.019**
**Splenic involvement** **Present (n=16)** **Absent (n=30)**	10 (62.5%) 8 (26.7%)	6 (37.5%) 22 (73.3%)	**0.020**	14 (%87.5) 24 (%80)	2 (12.5%) 6 (20%)	0.420	8 (50%) 4 (13.3%)	8 (50%) 26 (86.7%)	**0.010**	14 (87.5%) 16 (53.3%)	2 (12.5%) 14 (46.7%)	**0.020**	9 (56.3%) 3 (10%)	7 (43.8%) 27 (90%)	**0.001**	13 (81.3%) 9 (30%)	3 (18.8%) 21 (70%)	**0.001**
**Sedimentation rate (mm/h)** **>50 (n=17)** **≤50 (n=29)**	10 (58.8%) 8 (27.6%)	7 (41.2%) 21 (72.4%)	**0.038**	14 (82.4%) 24 (82.8%)	3 (17.6%) 5 (17.2%)	0.634	7 (41.2%) 5 (17.2%)	10 (58.8%) 24 (82.8%)	**0.077**	13 (76.5%) 17 (58.6%)	4 (23.5) 12 (41.4)	0.183	8 (47.1%) 4 (13.8%)	9 (52.9%) 25 (86.2%)	**0.017**	9 (52.9%) 13 (44.8%)	8 (47,1%) 16 (55,2%)	0.410
**EORTC** **Low-risk (n=11)** **High-risk (n=35)**	1 (9.1%) 17(48.6%)	10 (90.9%) 18 (51.4%)	**0.019**	7 (63.6%) 31 (88.6%)	4 (36.4%) 4 (11.4%	**0.079**	1 (9.1%) 11 (31.4%)	10 (90.9%) 24 (68.6%)	0.139	4 (36.4%) 26 (74.3%)	7 (63.6%) 9 (25.7%)	**0.028**	0 (0%) 12 (34.3%)	11 (100%) 23 (65.7%)	**0.021**	2 (18.2%) 20 (57.1%)	9 (81.8%) 15 (42.9%)	**0.026**
**IPS** **0-3 (n=39)** **≥4 (n=7)**	13 (33.3%) 5 (71.4%)	26 (66.7%) 2 (28.6)	**0.071**	31 (79.5%) 7 (100%)	8 (20.5%) 0 (0%)	0.236	8 (20.5%) 4 (57.1%)	31 (79.5%) 3 (42.9)	**0.064**	23 (59%) 7 (100%)	16 (41%) 0 (0%)	**0.038**	8 (20.5%) 4 (57.1%)	31 (79.5%) 3 (42.9%)	**0.064**	16 (41%) 6 (85.7%)	23 (59%) 1 (14.3%)	**0.037**
**HRS** **Rare (n=8)** **Medium/ abundant (n38)**	0 (0%) 18 (47.4%)	8 (100%) 20 (52.6%)	**0.012**	5 (62.5%) 33 (86.8)	3 (37.5%) 5 (13.2%)	0.129	0 (0%) 12 (31.6%)	8 (100%) 26 (68,4%)	**0.070**	3 (37.5%) 27 (71.1%)	5 (62.5%) 11 (28.9%)	**0.083**	0 (0%) 12 (31.6%)	8 (100%) 26 (68.4%)	0.161	17 (63%) 5 (26.3%)	10 (37%) 14 (73.7%)	**0.015**
**EBER** **Positive (n=27)** **Negative (n=19)**	13 (48.1%) 5 (26.3%)	14 (51.9%) 14 (73.7)	0.117	27 (100%) 11 (57.9%)	0 (0%) 8 (42.1%)	**<0.001**	10 (37%) 2 (10.5%)	17 (63%) 17 (89.5%)	**0.044**	22 (81.5%) 8 (42.1%)	5 (18.5%) 11 (57.9%)	**0.007**	9 (33.3%) 3 (15.8%)	18 (66.7%) 16 (84.2%)	0.161	17 (63%) 5 (26.3%)	10 (37%) 14 (73.7%)	**0.015**

**HRS:** Hodgkin/Reed Sternberg cells, **EBER:** Epstein-Barr virus-encoded RNA, **IPS:** International Prognostic Score.

## DISCUSSION

Macrophages are usually found in the microenvironment of solid tumors and hematologic malignancies (13). There has been increasing experimental evidence supporting the notion that macrophages promote tumor progression and facilitate cancer development (14–16). The relationship of TAM with B symptoms and a worse prognosis in cHL have been stated many years ago (8) and these findings were then supported by gene expression profiling studies. In their first gene expression profile study, Steidl et. al. have reported a decrease in progression-free survival when TAMs are more than 5%, by using CD68 immunohistochemistry (5). In a study conducted by Sanchez and colleagues with advanced-stage cHL patients from Spain and Houston, a significant correlation was found between high levels of CD68 expression (PG-M1 ve KP1 clones) and shortened duration of disease-specific survival in Spanish patients but no significant association was found with the patient group from Houston and with CD163 (11). This finding suggests that socio-demographic factors may also be effective in attaining different results in different studies. Similar to the above-mentioned studies, there are many other studies emphasizing that increased number of TAMs is associated with shortened overall survival, progression-free survival, and disease-free survival (4,17–27). While CD163 has been used either with CD68 PG-M1 (18), or CD68 KP1 (4,20,21,23) in some of these studies, only a single clone of CD68 has been utilized in others (17,19,22,24–27). A significant relationship was found between survival and both CD163 and CD68 expressions (4,20,21,23), or expression of either one of these markers (17–19,22,24–27). However, there are other studies which have found no relationship between survival and expression of either of these three markers (9,10). In our study, only increased expression of CD68 PG-M1 with the 40% cut-off point was found to be correlated with shortened overall survival. Also, there are many other studies stating a significant relationship between increased numbers of TAMs and clinicopathologic/laboratory parameters. These parameters include advanced (>45 years) age, male gender, presence of B symptoms, high IP score (>4 or>2), and advanced stage disease (4,5,17,20). In our study, increased numbers of TAM was found to be significantly related with the presence of B symptoms, splenic involvement, and high EORTC score. Like survival analyses, there are studies with opposite results in this respect (9). In our study, it was seen that CD163 positivity was higher than both clones of CD68 as in the literature (4,18,20). However, there are publications reporting the opposite (28). Correlation of the intensity of staining of all three markers found in other studies (20) and in this study suggests that using any marker while searching for TAM intensity may suffice, but the conflicting results in terms of significance suggest that it may be better to use CD163 and CD68 together. It is also unclear which clone of CD68 is more suitable for use. Since KP1 clone was used in most of the studies, significant results related to survival were obtained mostly with this clone (4,5,17–23). This fact reveals the need for other comparative studies. In assessing intensity of TAM with histiocytic markers in cHL, another issue is finding out the precise cut-off points. Different cut-off values have been used in other studies. While some have used the median value of expression rate (25%, 50%) of macrophage markers (17,18), some others preferred cut-off points determined in earlier studies (<5%, 5%-25%, 25%-50%, >50%) (9,11). As the expression levels obtained with CD163, CD68 PG-M1, and CD68 KP1 were sorted in a decreasing manner, the rates 50%, 40% and 30%, which were around the median values, were preferred as the primary cut-off values, respectively. To see the significance of lower levels of expression, rates of 30% for CD163 and 10% for CD68 PG-M1 and CD68 KP1 were determined as secondary cut-off values. EBV infection has been shown to alter the microenvironment of tumoral tissue in cHL patients (29), but it is controversial whether this infection has a prognostic role. Studies suggesting a shortened lifespan (9,20) are more common than others claiming that the survival period is not affected (4). No significant relationship between EBER positivity and overall survival was found in this study, although a borderline significance was obtained between with progression-free survival. Similar to some other reports, it was also found that TAM intensity determined by all three histiocytic markers was higher in EBER+ patients than in EBER– cases (4,9,11). This finding suggests that the presence of EBV may play an effective role in immune response and macrophage activation and polarization. Although this study was conducted with a relatively small group of patients and studies involving larger series with longer follow-up periods are needed, it was concluded that routinely examining TAM intensity in cHL patients and recording the results in pathology reports may not only provide additional prognostic information but may also contribute to future investigations.

## Conflict of INTEREST

The authors declare no conflict of interest.

## FUNDING

This work was supported by the Scientific Research Project Office of Manisa Celal Bayar University. Project Number: 2016-025.
